# Characteristics of Pediatric Crohn's Disease in Saudi Children: A Multicenter National Study

**DOI:** 10.1155/2016/7403129

**Published:** 2015-12-29

**Authors:** Omar I. Saadah, Mohammad El Mouzan, Mohammad Al Mofarreh, Ali Al Mehaidib, Mohammad Al Edreesi, Mohammed Hasosah, Abdulrahman Al-Hussaini, Khalid AlSaleem

**Affiliations:** ^1^Department of Pediatrics, Faculty of Medicine & Inflammatory Bowel Disease Research Group, King Abdulaziz University, P.O. Box 80205, Jeddah 21589, Saudi Arabia; ^2^Department of Pediatrics, Pediatric IBD Research Group, Prince Abdullah Bin Khalid Celiac Disease Research Chair, King Saud University, P.O. Box 2925, Riyadh 11461, Saudi Arabia; ^3^Department of Gastroenterology, Al-Mofarreh Polyclinic, P.O. Box 9789, Riyadh 11423, Saudi Arabia; ^4^Department of Pediatrics, Division of Gastroenterology, King Faisal Specialist Hospital and Research Center, P.O. Box 3354, Riyadh 11211, Saudi Arabia; ^5^Department of Pediatrics, Division of Gastroenterology, Dhahran Health Center, Saudi Aramco Medical Services Organization, P.O. Box 5000, Dhahran 31311, Saudi Arabia; ^6^Department of Pediatrics, Division of Gastroenterology, King Abdulaziz Medical City, National Guard Health Affairs, P.O. Box 8202, Jeddah 21482, Saudi Arabia; ^7^Division of Gastroenterology, King Fahad Medical City, Children's Hospital, King Saud bin Abdulaziz University for Health Sciences, P.O. Box 59046, Riyadh 11525, Saudi Arabia

## Abstract

*Background and Aims*. Crohn's disease (CD) is an evolving disease in KSA. Little is known about its characteristics in the Saudi population. The aims of this study were to describe the characteristics of Saudi children with CD and to determine whether the characteristics of CD in KSA are different from those seen in Western countries.* Methods*. In this study, children younger than eighteen years of age diagnosed with CD between January 2003 and December 2012 were included.* Results*. Of 330 patients identified, 186 (56.4%) were males. The median age at diagnosis was 15.8 years. A positive family history for IBD in first-degree relatives occurred in 13.6% of patients. The most common symptoms were abdominal pain (84.2%), weight loss (75.2%), and diarrhea (71.8%). The main disease location was ileocolonic (42.1%) and the main disease behavior was nonstricturing and nonpenetrating (63.6%). Perianal involvement was seen in 60 (18.2%) patients. Laboratory findings revealed anemia in 57.9% of patients, low albumin in 34.5%, and high CRP in 39.4%.* Conclusions*. Saudi children with CD have lower frequency of first-degree relatives with IBD, lower prevalence of early onset disease, longer diagnostic delay, higher prevalence of growth failure, and greater frequency of stricturing and penetrating disease behavior compared to Western patients.

## 1. Introduction

Inflammatory bowel disease (IBD) is a chronic immune-related disorder of the gastrointestinal tract, resulting in relapsing inflammation of the digestive tract. Crohn's disease (CD) and ulcerative colitis (UC) are the two main entities representing IBD. Whilst the inflammation in UC is restricted to the colonic mucosa, CD can involve any part of the gastrointestinal tract, affecting all layers and leading to bowel damage over time [1]. The underlying etiology remains largely unknown, but it may represent a complex interaction between genetic susceptibility, host immune response, and environmental factors [2]. CD with childhood onset represents 25% of all CD patients [3]. The childhood presentation is rather more extensive and severe as compared with adult presentation [4]. There has been a global trend of increasing incidence of CD over time in many countries [5]. In the Kingdom of Saudi Arabia (KSA), an incidence rate for CD of 0.27 per 100,000 individuals has been reported in children aged 0–14 years for the period between 2003 and 2012 [6]. Although the reported incidence is lower than that reported in Western countries, a significant increase in the trend is evident over time when two time periods are compared (2003–2007 and 2008–2012). In KSA, several small hospital-based case series have been published in children and adults with CD [7–13]. However, a comprehensive nationwide study is lacking. The aims of this collaborative multicenter study were to describe the clinical and laboratory characteristics of Saudi children with CD from different regions of KSA and to determine whether the characteristics of CD in KSA are different from those found in Western countries.

## 2. Patients and Methods

### 2.1. Study Description

This retrospective study involved children younger than eighteen years of age diagnosed with CD between January 1, 2003, and December 31, 2012. In KSA, children between birth and twelve years are exclusively managed by pediatric gastroenterologists, whereas those aged twelve to fourteen years are managed by either pediatric or adult gastroenterologists, depending on availability. Those aged fourteen to eighteen are managed mainly by adult gastroenterologists. Seventeen medical centers from different regions in KSA participated in this study. These centers are equipped with the necessary facilities to diagnose and manage IBD cases. Sixteen of the seventeen centers are government funded and provide free-of-charge services to all Saudi nationals and expatriate government employees, whereas one is a private center.

### 2.2. Study Subjects and Data Collection

Children with CD were identified either via the computerized hospital system using the ICD codes of the hospital admissions database, by endoscopy, or through personal physician records. A unified database collection form specifically designed for this study was completed by all participating centers and reviewed by gastroenterologists. The data collected included demographic, clinical, and laboratory information. The diagnosis of CD was made in accordance with ESPGHAN revised Porto Criteria [14]. Colonic infections and intestinal tuberculosis were excluded by appropriate investigations. The anatomical extent of CD was determined via the initial colonoscopy, upper endoscopy, and radiological studies. The disease location and behavior were defined according to the Paris classification [15].

Growth data were recorded on a spreadsheet with documentation of the date of measurements at diagnosis. Weight, height, and BMI were converted into *z*-scores using an anthropometric software program (Epi-Info, Centers for Disease Control and Prevention, Atlanta, GA, USA). Retarded growth parameters were defined as *z*-score values less than −2.0.

### 2.3. Statistical Analyses

Data were analyzed using SPSS 19 software (IBM Corp, Armonk, New York, United States). Descriptive data were reported as percentages of the total for categorical data, as means with standard deviations (SD), or as medians with interquartile range for numeric data.

### 2.4. Ethical Considerations

This study was approved by the Institutional Review Board of the College of Medicine (number 10/2647/IRB), King Saud University, Riyadh, KSA, as part of the research project entitled “Characteristics of inflammatory bowel disease in Saudi Children.”

## 3. Results

### 3.1. Demographic Data

**Figure 1:**
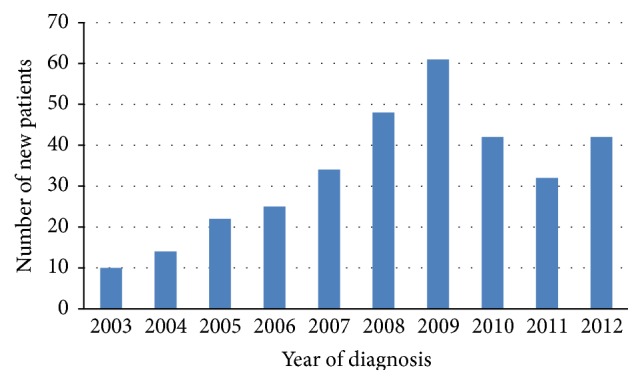
The number of new cases of CD diagnosed each year.

**Figure 2:**
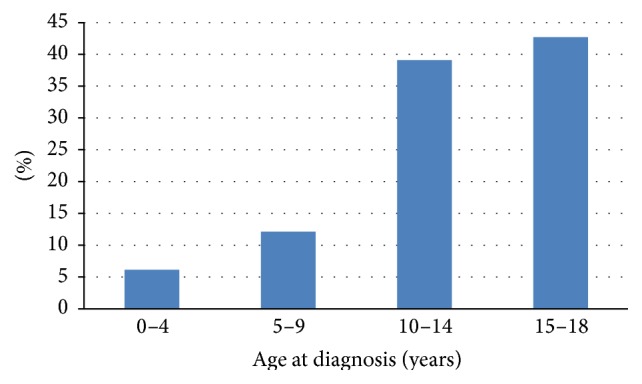
The age distribution pattern at the time of CD diagnosis.

From January 2003 to December 2012, 330 patients with CD were identified. The number of new cases in each year of the study period is shown in Figure 1, with the peak number being reported in the period between 2008 and 2010. In total, 186 patients (56.4%) were male and 144 (43.6%) were female. The median age at diagnosis was 15.8 years (range 0.2 to 18 years). The pattern indicating the number of cases distributed according to the different age categories is shown in Figure 2, which reveals that more patients were diagnosed after ten years of age. The median age at the onset of the symptoms was 13.8 years, with a median diagnostic delay of two years between the onset of the symptoms and the final diagnosis. The majority of patients (99.1%) were living in urban areas, whilst only three (0.9%) were from rural areas. They were distributed in twelve of the thirteen official country regions of KSA, with most patients residing in Riyadh (45.8%), followed by Makkah (21.2%) and the Eastern Province (17.9%). These three regions are identified as having the highest population density in KSA.

### 3.2. Family History

A positive family history for IBD in first-degree relatives occurred in forty-five patients (13.6%). Nineteen of the relatives (42.2%) had UC, twenty-four (53.3%) had CD, and two (4.4%) had unclassified IBD. The overall rate of consanguinity in this cohort was 35.8%, with a first cousin rate of 25.8% (*n* = 85).

### 3.3. Clinical Presentation

The presenting signs and symptoms are shown in Table 1. Of the 330 patients described in this cohort, 36 (10.9%) had other associated comorbidities, as shown in Table 2.

### 3.4. Growth

Analysis of growth data at diagnosis showed that 87 of 266 (32.7%) patients had a weight-for-age (WAZ) *z*-score of less than −2.0. The median weight-for-age *z*-score was −0.965 (range −13.9–6.8). The median height-for-age *z*-score was −0.645 (range −5.77–6.75) and 53 of 261 (20.3%) had height-for-age *z*-scores less than −2.0. BMI recording was available for 261 patients. The median BMI *z* score was −0.76 (range −10.4–2.9) and 77 (29.5%) patients had BMI *z*-score less than −2.0.

### 3.5. Disease Location and Behavior

The disease location and behavior at the time of diagnosis according to the Paris classification are shown in Table 3. Sixty (18.2%) patients had perianal involvement, ranging from isolated skin tags to severe perianal ulceration and fistulae. Perianal pathology included anal fistulae in twenty-six (7.9%) patients, skin tags in twenty-five (7.6%), anal fissures in twenty-three (7%), abscesses in eleven (3.3%), and ulcerations in five (1.5%) patients. Some patients had two or more combinations of different perianal pathology. Only patients with perianal fistulae, anal canal ulcers, and perianal abscesses were considered to have perianal disease modifiers according to the Paris classification (Table 3).

### 3.6. Laboratory Findings at Diagnosis

At diagnosis, 191 (57.9%) patients were anemic, with a mean (SD) hemoglobin level of 9.85 (1.49) g/dL (normal, 12–14.5 g/dL). Thrombocytosis was present in 140 (42.4%), with a mean (SD) platelet count of 662 (173) × 10^3^ /*μ*L (normal, 150–450 × 10^3^/*μ*L). One hundred and fourteen (34.5%) patients had low albumin with a mean (SD) albumin level of 23 (5.4) g/L (normal, 35–50 g/L). The erythrocyte sedimentation rate (ESR) was high in 184 (55.7%) patients, with a mean (SD) level of 53.8 (26.8) mm/hr (normal 0–15 mm/hr), whilst C-reactive protein (CRP) was high in 130 (39.4%) with a mean (SD) level of 40.9 (47.7) mg/L (normal, 0–3 mg/L). Serological testing for anti-*Saccharomyces cerevisiae* antibodies (ASCA) was positive in 47 (51%) out of ninety-two patients tested for IgA-ASCA and in 50 (54%) patients for IgG-ASCA, whilst perinuclear anti-neutrophil cytoplasmic antibody (p-ANCA) testing was positive in only ten (10.3%) of the ninety-seven patients tested for p-ANCA.

### 3.7. Therapy

Treatment was initiated at diagnosis with systemic steroids in 222 out of 228 patients (97.4%) in whom available information about the use of steroids was documented. A 5-amino salicylic acid derivative (Mesalazine) was used in 199 of 226 (88%) patients. The use of immunomodulators, most commonly Azathioprine, was documented in 188 out of 205 (91.7%) patients. Anti-tumor necrosis factor (TNF) antibodies in the form of intravenous Infliximab or subcutaneous Adalimumab were required for 70 and 22 patients, respectively. Data were missing regarding the use of enteral nutrition, evolution of treatment, and frequency of reported adverse effects.

### 3.8. Follow-Up and Complications

Patients were followed up and monitored by their treating physicians. The median duration of follow-up was 2.6 years (range 0.08 to 15.7 years). The clinical course of forty-six (13.9%) patients was complicated by the development of fistulae (*n* = 14), intestinal obstructions (*n* = 12), stricture formation (*n* = 9), abscess collection (*n* = 5), bowel perforation (*n* = 3), and central sinus thrombosis (*n* = 1). Seventeen (5.2%) patients were managed nonoperatively and twenty-nine (8.7%) required surgical intervention. Surgical procedures performed are shown in Table 4.

## 4. Discussion

Higher incidence rates for pediatric-onset CD are reported from the USA and Europe [16–19]. In Saudi Arabia, a lower incidence rate of CD (0.27 per 100,000 children) in children aged 0–14 years for the period between 2003 and 2012 was recently reported by our group [6]. Furthermore, an increase in the incidence of pediatric CD over time has been documented, in agreement with similarly observed trends reported from many countries [20, 21]. This is the first nationwide study that has included children with CD from different regions in the country. Saudi Arabia is located in the south-west corner of Asia, occupying 80% of the Arabian Peninsula, with an estimated surface area of 2,217,949 square kilometers. The country is divided into thirteen official country regions. According to the 2007 census, the total population of Saudi Arabia was 24 million but only 17.5 million were Saudi Arabian, of whom 32.5% were children between birth and fourteen years [22]. The majority of the population is distributed in the major cities in all regions, whilst smaller proportions are dwellers of the surrounding small towns and villages. The majority of our patients came from urban areas. Living in urban areas was considered to be among the risk factors for developing IBD, in agreement with the reported increasing risk associated with living in cities when compared with rural areas [23, 24]. We also found male predominance (56.4%) amongst the affected children with CD in our study at a similar level to the high male prevalence in various studies reported from different countries [4, 25, 26].

Early onset CD, defined as disease onset at or before five years of age, was seen in only twenty (6%) patients reported in our study. This was unexpectedly lower than the rates reported in studies from Japan, Europe, and North America [26–30]. Most of the patients who presented with early onset CD tended to have genetic diseases related to IL-10 and IL-10 receptor gene defects [31]. In the Saudi community, with its higher rate of consanguineous marriages, one would expect a significant proportion of this disease entity.

The median age at diagnosis of our patients was higher than reported in other countries [4, 17, 32]. Our data also indicated significant delay from the time of appearance of the first symptoms to the time of diagnosis (median delay of 24 months): this was longer than the median time reported in previous studies [33–35]. The median times to diagnosis reported from the Swiss IBD cohort, the prospective European cohort of patients with IBD (ECCO-Epicom cohort), and the French population-based cohort (EPIMAD) were 9, 4.6, and 3 months, respectively. This diagnostic delay may result in a proportion of patients being diagnosed after the age of eighteen years, meaning that they would not be eligible to be enrolled in this study despite having CD during adolescence. It might therefore be the case that the real proportion of children from birth to eighteen years with CD has been underestimated. The diagnostic delay may be influenced by many factors, such as the efficiency of the health care system, including referral procedures, the nonspecific nature of the presenting symptoms in CD, and the high prevalence of infectious diarrhea in our community. Efforts to reduce the diagnostic delay should include enhancement of public awareness, professional education of general pediatricians and family practitioners, and improvement to the referral system.

A notable finding in our study is the marked growth failure in terms of weight and height recorded in 32.7% and 20.3% of the participants, respectively. Growth failure evidenced by anthropometric measurements affects 10–40% of children with CD [36, 37]. In pediatric CD, growth may be affected by different contributing factors related to the disease. Growth failure may result from anorexia, malabsorption, intestinal inflammation, and corticosteroid usage [38]. The diagnostic delay observed in this study might allow enough time for the ongoing disease activity to negatively influence growth and hence contribute to the higher prevalence of growth failure.

In contrast to CD in adults, pediatric CD tends to have a high prevalence of extensive disease. The ileocolonic phenotype has been reported as being the most prevalent phenotype in 45.8–86% of pediatric patients with CD in a number of previous studies [27, 30, 32, 39–41]. In agreement with these findings, our study demonstrated the predominance of the ileocolonic phenotype in 42% of patients.

Studying the similarities and differences in the characteristics of pediatric Crohn's disease between developing and developed countries might help in understanding the pathogenesis of CD. Our data show many similarities to data from North America and Europe [19, 42, 43]: preponderance of males (56–62%), older age at time of diagnosis (peaking between 12 and 16 years of age), predominance of abdominal pain, diarrhea, weight loss, and blood in stool at presentation, the ileocolonic region being predominantly affected, and predominance of nonstricturing, nonpenetrating behavior. In contrast, the frequency of stricturing and penetrating behavior of CD is more frequent in Saudi children when compared with pediatric CD in Western countries (33% in our cohort versus 6–10% in Western data). This could be related to the significantly delayed diagnosis in our cohort. The frequency of family history of IBD in first-degree relatives in our data (13.6%) was lower than reported in North America [44] (30%). An overall consanguinity rate of 35.8% was found in our pediatric CD cohort. This is a very high rate compared to the rest of the world considering the overall prevalence of consanguinity in KSA of 56% [45].

Serological testing for ASCA-IgA, ASCA-IgG, and p-ANCA was positive in 51%, 54%, and 10.3% of our cohort, respectively, in agreement with the reported rate of detection of 40–60% for ASCA and 5–25% for p-ANCA antibody [46, 47]. Both ASCA and p-ANCA antibodies have been extensively studied in the literature. The ASCA antibody has been linked to CD, whilst atypical p-ANCA has been linked to the diagnosis of UC.

The most common therapeutic modality used at diagnosis to induce remission in this study was systemic steroid therapy (97%), followed by immunomodulators in 91% of cases and Mesalazine in 88%. These findings are comparable to the literature [48, 49]. The infrequent use of biologicals at diagnosis is consistent with the practice of the step-up approach used in many centers [50, 51].

It is important to address the study's limitations. The item in the database regarding treatment at diagnosis was completed only in 228 out of 330 patients (69%), probably because the information was not available in the medical records of some patients, especially those diagnosed in the earlier years of the study. This is a limitation associated with relying on information from medical records. This was a retrospective survey, conducted over a period of ten years, and the number of cases enrolled for analysis from all participating centers was small. Further, some cases that were under the care of adult gastroenterologists who were not participating in this study might have been missed. However, despite the abovementioned limitations, the study is noteworthy because it represents the contribution of almost all major centers dealing with the care of pediatric patients with CD in Saudi Arabia.

In conclusion, the characteristics of Saudi pediatric patients with CD differ from those in the West in many aspects, including higher rate of consanguinity, lower frequency of first-degree relatives with IBD, lower prevalence of early onset disease, greater diagnostic delay, higher prevalence of growth failure, and higher frequency of stricturing and penetrating disease behavior than those reported in the Western literature. The similarities include older age at diagnosis, predominance of male gender, similar main presenting symptoms, predominance of the ileocolonic phenotype, and predominance of nonstricturing, nonpenetrating disease behavior. Further collaborative prospective studies will be important to identify the risk factors, the social and economic impact, and the natural history of the disease in the Saudi community.

## Figures and Tables

**Table 1:** The presenting symptoms and signs of children with Crohn's disease (*n* = 330).

	Number	Percentage
Symptoms		
Abdominal pain	278	84.2
Weight loss reported	248	75.2
Diarrhea	237	71.8
Anorexia	130	39.4
Bleeding per rectum	104	31.5
Vomiting	82	24.8
Bloating/flatulence	75	22.7
Fever	68	20.6
Nausea	47	14.2
Altered bowel habits	27	8.2
Perianal disease	27	13
Joint pain	30	9.1
Constipation	21	6.4
Tenesmus	19	5.8
Mouth sore	9	2.7
Rash	6	1.8
Clinical signs		
General manifestation		
Underweight (below 5th percentile)	193	58.5
Overweight	1	0.3
Mouth ulcers	5	1.5
Clubbing	2	0.6
Generalized edema	2	0.6
Skin examination		
Erythema nodosum	3	0.9
Pyoderma gangrenosum	3	0.9
Ichthyosis	1	0.3
Hyperpigmentation	1	0.3
Hypopigmentation	1	0.3
Abdominal examination		
Abdominal tenderness	117	35.4
Abdominal mass	10	3
Abdominal distention	3	0.9
Hepatosplenomegaly	2	0.6
Ascites	1	0.3

**Table 2:** Associated comorbidity in 36 patients with Crohn's disease.

	Number	Percentage
Respiratory system		
Bronchial asthma	5	14
Chronic lung disease	1	2.8
Central nervous system		
Seizure disorders	2	5.6
Tuberous sclerosis	1	2.8
Acquired demyelinating neuropathy	1	2.8
Nystagmus-albinism (Hermensky-Pudlak Syndrome)	1	2.8
Hematology		
Glucose-6-phosphate dehydrogenase deficiency	4	11
Skin		
Epidermolysis bullosa	1	2.8
Renal system		
Nephrotic syndrome	2	5.6
Chronic glomerulonephritis	1	2.8
Endocrine system		
Hyperthyroidism	1	2.8
Hypothyroidism	1	2.8
Gastrointestinal system		
Autoimmune hepatitis	1	2.8
Celiac disease	1	2.8
Rheumatology		
Kawasaki disease	1	2.8
Arthritis	3	8.3

**Table 3:** Disease location and behavior at presentation in 330 patients with Crohn's disease according to Paris classification.

	Number	Percentage
Location		
Distal ileum (L1)	93	28.2
Colonic (L2)	53	16.1
Ileocolonic (L3)	139	42.1
Upper disease proximal to ligament of Treitz (L4a)	31	9.4
Upper disease distal to ligament of Treitz and proximal to the distal 1/3 ileum (L4b)	14	4.2
Behavior		
Nonstricturing nonpenetrating (B1)	210	63.6
Stricturing (B2)	64	19.4
Penetrating (B3)	20	6.1
Penetrating and stricturing (B2B3)	26	7.9
Perianal disease modifier (p)	42	12.7

**Table 4:** Surgical procedures (*n* = 31) performed for 29 Crohn's patients with complications.

	Number	Percentage
Ileocecal resection	11	37.9
Ileal resection	5	17.2
Fistulectomy	4	13.8
Subtotal colectomy	3	10.3
Hemicolectomy	2	6.9
Abscess drainage	3	10.3
Ileostomy	1	3.4
Cecostomy	1	3.4
Appendectomy	1	3.4
